# Time on task limits psychotherapy's role in reducing the societal burden of aggression

**DOI:** 10.3389/fpubh.2025.1570642

**Published:** 2025-05-08

**Authors:** Joseph Strayhorn, Stephen V. Faraone, Yanli Zhang-James

**Affiliations:** ^1^Department of Psychiatry and Behavioral Sciences, Norton College of Medicine at SUNY Upstate Medical University, Syracuse, NY, United States; ^2^Clinical Psychology Psychiatry and Behavioral Sciences, Norton College of Medicine at SUNY Upstate Medical University, Syracuse, NY, United States; ^3^Department of Neuroscience and Physiology, Norton College of Medicine at SUNY Upstate Medical University, Institute for Human Performance, Syracuse, NY, United States

**Keywords:** intermittent explosive disorder, psychotherapy utilization, impulsive anger, deliberate practice, time on task, aggression, anger control, societal burden

## Abstract

**Introduction:**

Aggression and violence, people's inhumanity to one another, are perhaps society's foremost problems. One approach to this problem is the provision of traditional clinical services through psychotherapy. Anger control is a learnable skill, but such learning requires “time on task.” Our goal was to shed light on the potential impact of psychotherapy as a public health remedy, by studying how much psychotherapeutic intervention is being delivered to patients with impulsive aggression classified as Intermittent Explosive Disorder (IED).

**Method:**

Using de-identified electronic health record data from TriNetX, collected from 87 medical institutions, we analyzed the distribution of psychotherapeutic sessions received by 32,322 individuals with IED.

**Results:**

The distribution of psychotherapeutic sessions is highly skewed, resembling a curve of inverse proportion. The mode and the median for sessions attended were zero; the mean was four sessions. Only about 25% of patients received any psychotherapy. Approximately 10% attended nine visits or more; 5% 30 or more; 2% 50 or more. Eighty percent of the psychotherapeutic labor went to the 7.5% of patients who could attend over 14 sessions; about half the psychotherapeutic labor went to the 2.5% of patients who could attend 40 or more sessions. Thus, a small subset of patients absorbed most of the psychotherapeutic labor, and most patients did not spend enough (or even any) psychotherapeutic time on task.

**Discussion:**

Traditional psychotherapy delivered through health care systems appears to deliver sufficient “time on task” to only a small subset of individuals with impulsive aggression. Multipronged public health solutions to aggression and violence must be pursued by society as a whole. The efforts of mental health professionals are important and necessary, but the job should not be delegated to clinicians alone.

## Introduction

Aggression, violence, maladaptive anger, and “man's inhumanity to man” constitute what is arguably the foremost problem of the human species. Globally, almost half a million people died from homicide in 2021, not counting those perishing from warfare (1). The World Health Organization lists interpersonal violence as the second most frequent cause of mortality in the world's 15–19 year olds (2). In the US, about 40% of people report being afraid to walk alone at night in regions within a mile of their residence (3). Forty percentage of US K-12 teachers report experiencing physical violence from students, and 68% have experienced verbal abuse (4). Of over 336,000 women in 21 countries, 37% reported experiencing intimate partner violence (5). Fearing one's fellow human beings appears to exacerbate all mental health disorders. Aggression in the form of nuclear warfare threatens human civilization itself.

What can be done about the public health problem of aggression and violence? One approach, frequently mentioned after highly publicized violent incidents, is to increase the availability of traditional clinical mental health services. Within traditional mental health services, by far the most common interventions are medications and psychotherapy. A discussion of drug therapy for aggression is beyond the scope of this article, except to comment that the US Food and Drug Administration has yet to approve a drug for the general treatment of aggressive behavior (6), although two antipsychotics are approved for irritability associated with autism (7).

Psychotherapy and psychoeducation for anger and aggression have been widely studied: at least 21 meta-analyses of such methods have been published (8). But psychotherapy requires the investment of time and effort, both by therapist and client. How frequently does sufficient energy get directed toward this problem? “Time on task,” “engaged time,” and “deliberate practice” are major topics in the education literature (9, 10), but infrequently addressed in the mental health literature.

The definitive way to determine how many hours are necessary would be a mastery learning approach: to deliver training to large numbers of people of carefully measured characteristics until sustained remission from aggression is reached. Such a strategy, unsurprisingly, has never been employed.

Another strategy is to guess time requirements based on the complexity of the skills involved and the time required for learning other complex skills. To attain the highest level of proficiency in violin, 7,000–10,000 h of deliberate practice are usually required (11). Aspiring concert piano students often devote about 1,400 h per year during the teen years (12). Among competitive high school swimmers, “All but one or two of our subjects were swimming 4 h (or more) a day, six and sometimes seven times a week. During the summer, even more time was spent in practice.” This translates to at least 1,250 h per year; similar time investments were found for experts in mathematics, tennis, and research in neurology (12). The US State department estimates that for an English-speaking adult to learn “general professional proficiency” takes 600 to 750 class hours for Spanish and 2,200 h for Japanese or Mandarin (13). A US K-12 student spends about 1,227 h in school, per grade (14). A 3 credit college course is expected to require 135 h (15). Required practice driving, in New York State, for those with learners' permits before taking a road test is 50 h (16). Although these skills differ substantially in nature from those developed for anger control through psychotherapy and psychoeducation, these examples highlight significant time investments required for learning complex skills, underscoring the importance of long-term engagement and commitment.

If habits that are stable over time are more resistant to change, aggression is one of those: a study measuring anger of children longitudinally in first, third, and fifth grade reported that “effect sizes for anger stability were substantial, with stability correlations for consecutive assessments ranged from 0.55 to 0.70” (17).

The skill set relevant to non-aggressive functioning is large and complex. The treatment manual for Aggression Replacement Training, a widely used program (18), lists a “skill streaming checklist” comprising 50 skills, for example answering a complaint, being a good sport, responding to failure, dealing with an accusation, and dealing with group pressure. For each of the 50 skills, the manual lists three to five steps in execution of the skill. It is very plausible that for some learners, practicing for 1 h one each step would be just the beginning of attaining mastery; this would necessitate about 150 h of time on task.

Especially for children, since low reading skill can make school a daily source of frustration, reading is an anti-aggression skill (19–23). Patterson and colleagues reported that an average of 33 h of one-to-one work resulted in an improvement of one grade equivalent (23).

A sampling of manuals on anger control (23–28) reveals many component skills that could be included in psychoeducation or psychotherapy for aggression. Covering the content of such manuals, along with practice of the skills, would appear to be a very ambitious task for a course consuming 135 h. It is quite plausible that certain learners would need hours on task numbering over 135, or in the hundreds, or even in the thousands, to achieve mastery in the skills of anger control and non-violence. And given the often disastrous life consequences of very poor levels of such skills, the devotion of such hours, if necessary and successful, would be time well spent.

How much time has been devoted to anger control research interventions? Researchers face constraints: the greater the training time, the greater is the dropout rate, the longer the comparison group goes without the presumably useful intervention, and the longer the study takes. In a meta-analysis of studies of psychological treatments for anger (29), the mean number of treatment sessions was as 8.5 (SD 3.72), with range from 3 to 40. The number of sessions was positively correlated with effect size. Determining requisite time for learning anger control skills is complex because the yield of effort depends so exquisitely upon baseline characteristics of the research sample. McCloskey et al. (30) reported favorable effect sizes for a 12 sessions, 10 h intervention; Larden et al. (31) found no effect from a program which encompasses about 45 h. The first of these studied adult outpatients who voluntarily signed up for the intervention and self-reported their outcomes; the second was with adult convicted violent offenders who began training in the prison system, for whom the outcome measure was violent recidivism rate.

How much time do people with aggression problems spend learning psychological skills in non-research clinical settings? The US National Comorbidity Study Replication studied utilization of services over 12 months by people meeting criteria for mental health diagnoses. Among these was Intermittent Explosive Disorder (IED), a pattern of recurrent impulsive aggressive outbursts and the only DSM diagnosis where impulsive aggression *must* be present (32). Only 13.9% of those with IED obtained any treatment from a mental health service provider (33). This was the lowest rate of service utilization of all the diagnostic categories studied. Of those who did see a mental health services provider, the median number of visits was 3.5 sessions. The adolescent supplement of that study (34) reported that only 6.5% of adolescents with IED obtained specific treatment for anger over 12 months.

The current study sheds more light upon how many sessions of psychotherapy people with IED actually attend.

## Methods

Our study uses data on patients diagnosed with Intermittent Explosive Disorder (IED). IED is certainly not the only DSM diagnosis where aggression, anger, irritability, or violence can be a prominent symptom; it is, however, the only one where impulsive aggression *must* be present (32). Our data set was obtained from the TriNetX Research Network (35), which contained de-identified data from 117.7 million patients across 87 healthcare organizations (HCOs) globally at the time of the study (January 31, 2024). Among them, 33,547 patients had at least one diagnostic code for IED (ICD-10-CM F63.81, or ICD-9-CM 312.34 and 321.35). Because the data contains only de-identified patient medical records, the study was determined to be exempt by the SUNY Upstate Medical University Institutional Review Board.

We tallied the number of psychotherapeutic sessions for individuals diagnosed with this disorder. Because the distributions were very similar for adults and minors, we combined the subsamples for the analyses we report here.

We excluded patients with missing years of birth and those from healthcare organizations outside the US. We also excluded those who were diagnosed with IED or had their first healthcare encounter on or after January 1st, 2023, in order to allow sufficient time to engage in therapies. We excluded children with diagnosis recorded at 5 years of age or younger, since the DSM 5 requires attainment of age 6 for diagnosis. After these exclusions, 32,322 people remained in the sample diagnosed with Intermittent Explosive Disorder.

Psychotherapy sessions were identified using the Current Procedural Terminology (CPT^®^) codes 90832, 90833, 90834, 90836, 90837, 90838, 90839, 90840, 90845, 90846, 90847, 90849, and 90853. Discontinued CPT codes used prior to 2013 for psychotherapy sessions were fully mapped to the current codes. In the rare event where more than one code was entered on the same day, these were counted as one session. The psychotherapy sessions were counted for each patient. For each number of sessions, the total number of patients who had been seen that many times was tabulated. Extraction and preprocessing of psychotherapeutic sessions were conducted using STATA Version 18 (36) (Version 18; StataCorp LLC, College Station, TX). Histograms, graph of cumulative fraction of patients by number of visits, and Lorenz curve (cumulative fraction of visits by cumulative fraction of patients) were generated with R, Version 4.4.2 (37).

## Results

### Sample characteristics

A total of 32,322 patients with IED diagnoses were included in the analysis. Of these, 71.4% were male, 25.2% were female, and 3.3% had unknown sex. The racial distribution was as follows: 64.5% were White, 14.7% were Black or African American, 1.1% were Asian, 0.6% were American Indian or Alaska Native, 0.4% were Native Hawaiian or other Pacific Islander, and 18.8% were of unknown or other races. Additionally, 10.1% of patients were Hispanic or Latino, 65.7% were non-Hispanic, and 24.2% had unknown ethnicity. The mean age of the patients was 35.4 years, with a standard deviation of 17.6 years. The mean age at IED diagnosis was 25.5 years, with a standard deviation of 17.1 years. At the time of their first visit, 60.8% of patients were 18 years or older, while 39.2% were under 18. Medical records of psychotherapy spanned from 1985 to 2023, with median of 2018, an interquartile range (IQR) from 2015 – 2021, and 1st to 99th percentile from 1998–2023. Median year of diagnosis of IED was 2017 (IQR: 2013 – 2020, 1st and 99th percentile: 1995–2022), suggesting most of the diagnoses were likely based on DSM-IV and DSM-5 criteria.

### The distribution of psychotherapeutic effort

Table 1 summarizes the number of people who had each number of sessions. It also displays cumulative percent frequencies for the number of people, and for the number of visits.

**Table 1:** Distribution of number of sessions, full sample.

**N of Sessions**	**Frequency for *N***	**Cumulative fraction people**	**Visits for this *N***	**Cumulative fraction visits**
0	24,214	0.749	0	0.000
1	1,446	0.794	1,446	0.011
2	868	0.821	1,736	0.025
3	616	0.840	1,848	0.039
4	489	0.855	1,956	0.054
5	401	0.867	2,005	0.069
6	311	0.877	1,866	0.084
7	287	0.886	2,009	0.099
8	226	0.893	1,808	0.113
9	224	0.900	2,016	0.129
10	191	0.906	1,910	0.144
11	179	0.911	1,969	0.159
12	167	0.916	2,004	0.174
13	143	0.921	1,859	0.189
14	143	0.925	2,002	0.204
15	135	0.929	2,025	0.220
16	116	0.933	1,856	0.234
17	105	0.936	1,785	0.248
18	112	0.940	2,016	0.264
19	88	0.942	1,672	0.277
20	105	0.946	2,100	0.293
21–30	643	0.966	15,718	0.414
31–40	323	0.976	11,286	0.501
41–50	210	0.982	9,493	0.575
51–60	140	0.986	7,682	0.634
61–70	108	0.990	7,061	0.689
71–80	71	0.992	5,312	0.730
81–90	65	0.994	5,559	0.773
91–100	43	0.995	4,082	0.804
101–200	116	0.999	14,974	0.920
201–300	28	1.000	6,757	0.972
301–400	7	1.000	2,571	0.992
401–500	1	1.000	456	0.995
501–600	1	1.000	590	1.000
	Total Pts 32,322		Total Visits 129,429	

In the total sample, both the median and the mode for psychotherapy visits were zero, since 75% did not receive any psychotherapy. Across the entire cohort, there were 129,482 psychotherapy visits in total, resulting in an average of 4.0 visits per person when considering the whole sample. Notably, only about 9% of the total cohort received at least 12 visits, the threshold used in the McCloskey et al. (30) study of psychotherapy for Intermittent Explosive Disorder.

When focusing specifically on the subset of the patients who received any psychotherapy at all (25% of the sample, or 8,108 individuals), the most common number of sessions (mode) was 1. The median number of sessions in this treated group was 5, while the average was 16. Among those who engaged in psychotherapy, about 16% completed 12 or more sessions.

A small fraction of the sample devoted the time on task that would appear plausibly required to alleviate the symptoms of Intermittent Explosive Disorder. Only 10% of the sample received more than 10 sessions. Less than 4% completed as many as the 30 sessions employed by Aggression Replacement Training (18). About 2% had 50 sessions or more.

Who received the nearly 130,000 psychotherapy sessions? A large fraction of the psychotherapeutic labor went to a small fraction of the patients. Eighty percent of sessions went to the 7.5% of patients who could attend 14 sessions or more. Fifty percent of the psychotherapeutic labor went to the 2.5% of the patients who could attend more than 40 sessions. Twenty percent of the labor went to the 0.5% of patients who could attend more than 100 sessions (these numbers are rounded). Thus, the picture is of therapists who are doing lots of work, but they are doing it with a small fraction of the total patient sample.

**Figure 1:**
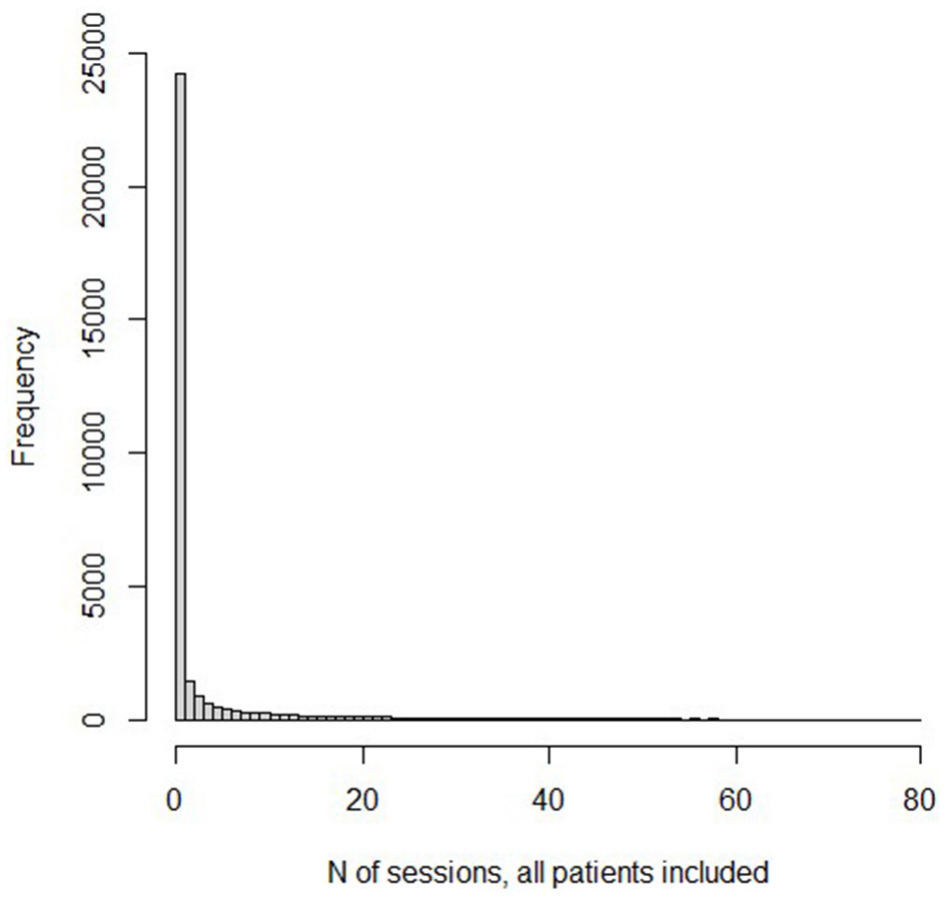
Histogram of *N* of sessions, all patients included.

**Figure 2:**
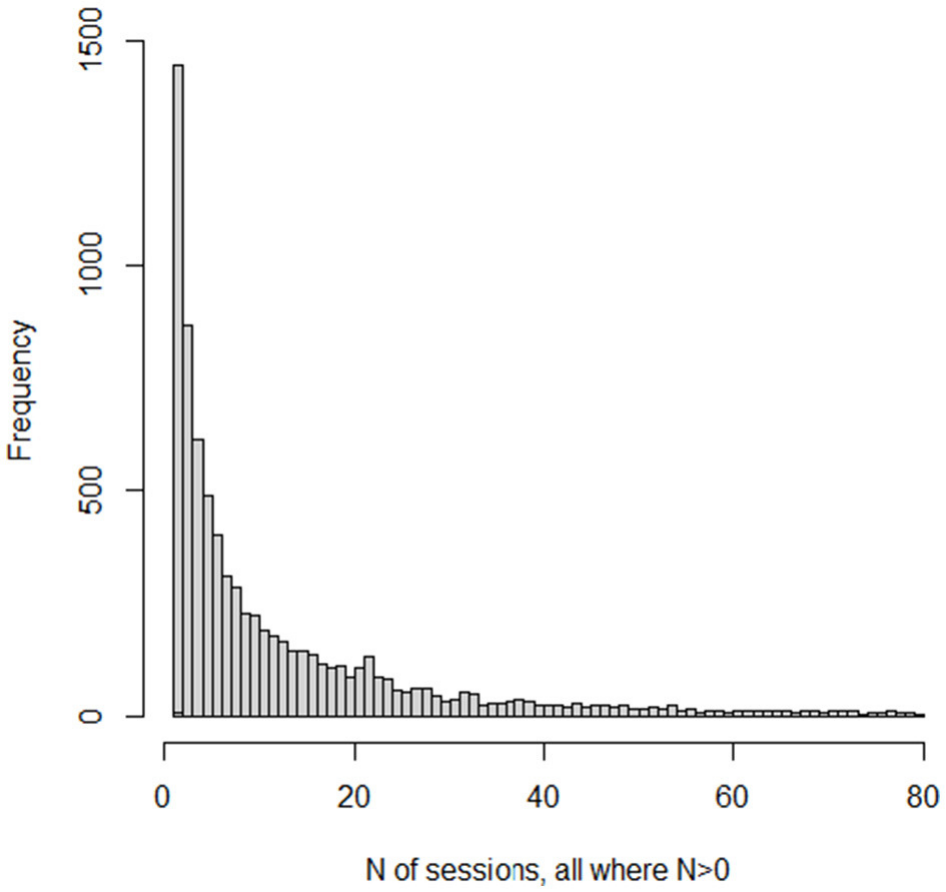
Histogram of *N* of sessions, all where *N* > 0.

**Figure 3:**
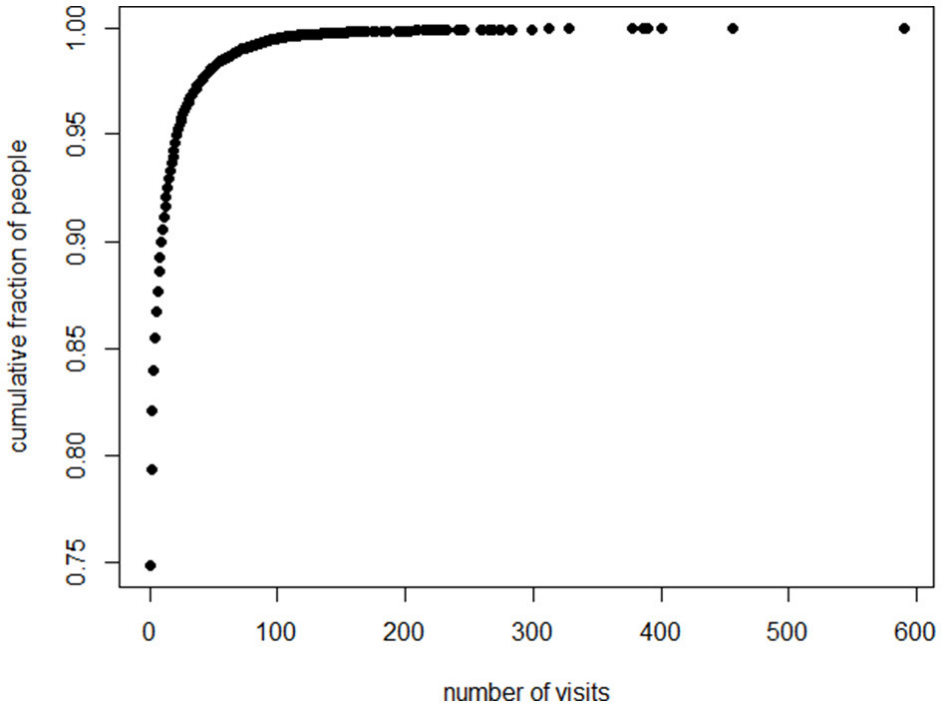
Cumulative distribution, number of people with numbers of visits.

**Figure 4:**
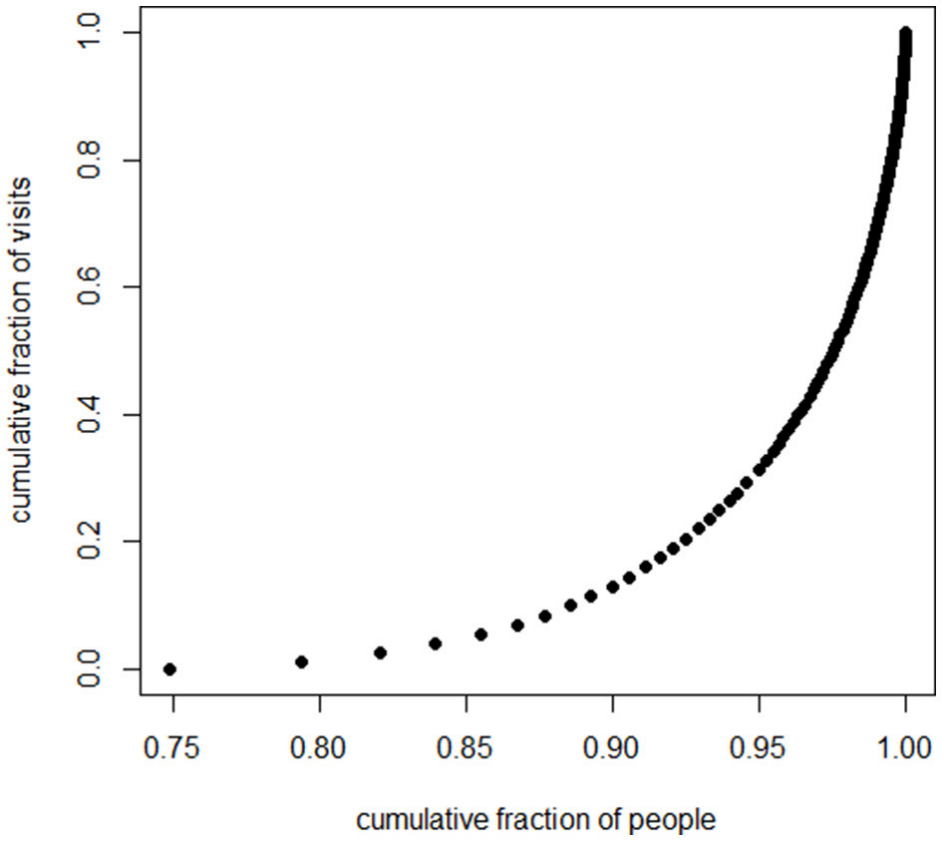
Lorenz curve, people and visits.

Several graphs picture the distribution. Figure 1 displays a histogram for the frequencies for the numbers of sessions. Because the number of “0” sessions is so large, we also present in Figure 2, a histogram for the subset of patients who had at least one psychotherapy session. Because the frequencies past 80 sessions are too small to register on the graphs, the histograms are truncated at 80 sessions. The graph of the cumulative fraction of the sample who came for a given number of visits is as in Figure 3. A plot of the cumulative fraction of individuals vs. the cumulative fraction of visits (called a Lorenz curve) yields the curve in Figure 4.

The distributions of number of psychotherapy sessions do not even slightly resemble normal distributions. Rather, they resemble the shape of an inverse function, y = 1/x. The number of patients completing visits is an almost monotonically decreasing function of the number of visits. This is also the general shape of a form of the Pareto Distribution (38). The Pareto function, as well as the inverse function, yields linear results when the logarithm of the dependent variable is plotted vs. the logarithm of the independent variable. Several analyses and graphs not included here supported the linearity of the log-log relation.

## Discussion

Our results reveal two striking findings. First, the total number of sessions (and thus, time on task) in psychotherapy, for the vast majority of our sample, appears to fall well below the range that may produce clinically meaningful improvements, as suggested by prior research (29–31). While our dataset does not include direct clinical outcomes, this low number of sessions raises concerns about the likelihood of achieving therapeutic benefits consistent with established evidence. Second, a large fraction of the psychotherapeutic resources spent on patients with IED is delivered to a small subset of the people.

A habit of impulsive aggression can be devastating to the life trajectory not only of the patient, but also of the recipients of the aggression. If the amount of time supposedly spent on a 3 credit college course (135 h) were to yield much better results than much smaller time investments, the larger efforts would unquestionably be worthwhile; however, as far as we can find, there are no data on how much could be achieved by those levels of time on task or higher.

With distributions of the shape presented here, doubling or tripling the labor pool of psychotherapists would still leave vast numbers of people with substantial aggression problems undertreated or not treated at all. If all the patients in the population with IED were to receive adequate psychotherapeutic treatment, it is very likely that this would “break the bank” of third party payers.

A great deal of work has been done in designing and testing psychotherapeutic interventions for aggressive behavior. But for the vast majority of individuals diagnosed with IED in our sample, the information collected in the studies cited in 21 meta-analyses of psychotherapy for aggression (8) was unfortunately irrelevant. For the vast majority, there was either no time or very little time devoted to psychotherapy.

These results would imply that public health efforts to reduce the societal problem of aggression should not be confined to screenings for, referrals to, and conduct of, psychotherapy. They suggest that the problem is too big to be solved by clinical professionals alone. There is not a shortage of other ideas. A few examples include voluntary reduction of media models of violence (39); large scale psychoeducational programs in schools and elsewhere (40); including anti-bullying programs (41); eradicating lead toxicity (42); discouraging alcohol intake (43); reduction of availability of guns and other weapons (44, 45); reduction of poverty (46, 47); promoting international dispute-resolution methods (48); and others. And surely new ideas and efforts will arise if we avoid the mentality that maladaptive aggression can only be treated and prevented by highly trained and licensed specialists.

Our conclusions reinforce those of Kazdin and Blase (49), who have examined the constraints on providing enough psychotherapy to relieve the societal burden of mental illness and have called for a “rebooting” of research and practice. They stated:

“Most people who might benefit from services for their dysfunction do not receive care. Additional resources in terms of person power might help. However, the dominant model of treatment delivery in clinical practice focuses on in-person treatment provided to individuals or relatively small units (groups, family, and couples). The model constrains the ability to reach individuals in need, even if the number of mental health professionals doubles.” (p. 33)

A limitation of this study is that we have no way of counting the amount of time on task that patients spent in homework between sessions. Although it is possible that some of them may have received a few hours of training which they supplemented by numerous hours of independent work at home, we are not optimistic enough to imagine that this takes place very frequently.

A limitation that could possibly lead our results to be understated is that the people with an Intermittent Explosive Disorder diagnosis recorded in our sample represent only about 0.027% of the total pool of patients from which we drew, even after adjustment for the fraction eliminated from our sample. This represents a small fraction of the estimated prevalence of Intermittent Explosive Disorder in the population (50, 51). If this is because clinicians set a high bar for assigning the diagnosis, and have selected the most aggressive individuals, or the ones most desiring help for aggression, we might guess that the investment of time on task could be even lower for impulsively aggressive individuals not diagnosed. Our results on the sparsity of psychotherapeutic intervention are consistent with those of National Comorbidity surveys mentioned above, (33, 34) which gave an impression of even fewer psychotherapy sessions devoted to Intermittent Explosive Disorder than our results do.

Another limitation implying that our results are understated is as follows. The visits we counted represent an upper limit that almost certainly overestimates the sessions actually devoted to the topic of aggression. In the same sample, Intermittent Explosive Disorder was found to be comorbid with almost all other psychiatric conditions (52). It is very likely that psychotherapy visits were devoted to anxiety, depression, and other comorbid conditions, in addition to those devoted to anger control.

Our results, of course, do not answer the “why” question: why is psychotherapy utilization so low and unequal? Aggression as a source of power, the conflict of psychotherapy hours with work and school time, full caseloads for therapists, and lack of financial coverage or access to service and therapist are all hypotheses to be considered. Stigma surrounding mental health, including societal judgment and internalized shame, often leads to the underutilization of psychotherapy. Fear of being labeled or misunderstood can discourage individuals from seeking help, even when they recognize the need for treatment. Another possibility is that people with impulsive aggression often have a personality orientation toward enacting rather than reflecting; this may make sitting and reflecting in psychotherapy sessions unpleasant. Our guess is that many of the factors involved will not be easy to change.

The conclusion of this analysis in no way diminishes the value and worth of psychotherapy for aggression, both directly, and in providing learnings informing the content of more widely scalable psychoeducational interventions. Increasing the availability of psychotherapy is undoubtedly a positive goal. But the analyses we present constitute a call to action for much creative work in devising and implementing public health approaches to the problem, over and above those of traditional clinical practice. Remedies for the public health problem of aggression are the bailiwick of all society, not just mental health professionals.

## Data Availability

The datasets presented in this article are not readily available because the individual-level electronic health record data in the TriNetX database cannot be shared, as they are the property of TriNetX and can only be accessed through direct contract with the company. Summary data and results are provided in the Table and Figures provided in the manuscript. Further inquiries can be directed to the corresponding author.
